# NKT Cell Responses to B Cell Lymphoma

**DOI:** 10.3390/medsci2020082

**Published:** 2014-04-14

**Authors:** Junxin Li, Wenji Sun, Priyanka B. Subrahmanyam, Carly Page, Kenisha M. Younger, Irina V. Tiper, Matthew Frieman, Amy S. Kimball, Tonya J. Webb

**Affiliations:** 1 Department of Microbiology and Immunology, University of Maryland School of Medicine, Baltimore, MD 21201, USA; 2 Department of Medicine and the Marlene and Stewart Greenebaum Cancer Center, University of Maryland School of Medicine, Baltimore, MD 21201, USA

**Keywords:** NKT cells, CD1d, mouse models of lymphoma, mantle cell lymphoma, α-galactosylceramide

## Abstract

Natural killer T (NKT) cells are a unique subset of CD1d-restricted T lymphocytes that express characteristics of both T cells and natural killer cells. NKT cells mediate tumor immune-surveillance; however, NKT cells are numerically reduced and functionally impaired in lymphoma patients. Many hematologic malignancies express CD1d molecules and co-stimulatory proteins needed to induce anti-tumor immunity by NKT cells, yet most tumors are poorly immunogenic. In this study, we sought to investigate NKT cell responses to B cell lymphoma. In the presence of exogenous antigen, both mouse and human NKT cell lines produce cytokines following stimulation by B cell lymphoma lines. NKT cell populations were examined *ex vivo* in mouse models of spontaneous B cell lymphoma, and it was found that during early stages, NKT cell responses were enhanced in lymphoma-bearing animals compared to disease-free animals. In contrast, in lymphoma-bearing animals with splenomegaly and lymphadenopathy, NKT cells were functionally impaired. In a mouse model of blastoid variant mantle cell lymphoma, treatment of tumor-bearing mice with a potent NKT cell agonist, α-galactosylceramide (α-GalCer), resulted in a significant decrease in disease pathology. *Ex vivo* studies demonstrated that NKT cells from α-GalCer treated mice produced IFN-γ following α-GalCer restimulation, unlike NKT cells from vehicle-control treated mice. These data demonstrate an important role for NKT cells in the immune response to an aggressive hematologic malignancy like mantle cell lymphoma.

## 1. Introduction

Non-Hodgkin’s lymphomas (NHL) are one of the few cancers of which incidence has increased over the past thirty years. NHL are a large heterogeneous group lymphomas, of which >80% arise from B cells. Diffuse large B-cell lymphoma (DLBCL) is the most common subtype; it is an aggressive lymphoma with heterogeneous clinical behaviors. DLBCL accounts for 25%–30% of NHL among adults in the United States and although 60% of patients respond well to current therapy and have prolonged survival, the remainder succumb to the disease [1]. Another subtype of NHL, mantle cell lymphoma (MCL) is an aggressive disease that is characterized by the abnormal accumulation of CD20^+^CD5^+^ B cells in the lymph nodes, spleen, bone marrow, and blood. Although treatment with combination chemotherapy can be effective, most patients relapse, and the outcome for MCL remains poor with a median survival of only five years [2–4]. Thus, novel approaches for the treatment of both DLBCL and MCL are essential.

Clinical studies have demonstrated that while immunotherapy can effectively treat lymphoma, many patients relapse [2–4]. New strategies that focus on restoring the host’s anti-tumor immune responses are needed to improve therapeutic outcomes (as reviewed in [5–9]). Many studies have demonstrated that the host’s immune system has critical functions, such as cancer immune surveillance, and can recognize neoplastic transformation and destroy malignant cells (as reviewed in [10,11]). This is a multifaceted process where the first recognition events are contributed by early, innate immunity. These events lead to the activation of the adaptive immune system, which results in the effective clearance of tumor cells.

Natural killer T (NKT) cells are a unique subset of lymphocytes that recognize lipid antigens in the context of CD1d, a non-classical MHC class I molecule and serve as a link between the innate and adaptive immune system through their expeditious release of a number of different cytokines (as reviewed in [12–14]). There are two defined subsets of NKT cells. Type I NKT cells (also known as invariant NKT cells, or *i*NKT cells) express a semi-invariant Vα14Jα18 TCR in mice and Vα24Jα18 TCR in humans [15–18]. Type II NKT cells are CD1d restricted T cells that express a more diverse set of α chains in their TCR. The two types of NKT cells often exert opposing effects, especially in tumor immunity, where type II cells generally suppress tumor immunity, while type I NKT cells enhance anti-tumor immune responses [19]. In addition to playing an important regulatory role in tumor surveillance [20,21], *i*NKT cells have been demonstrated to play a role in autoimmune disease [22], infectious disease, and inflammatory conditions, such as ischemia reperfusion injury [23,24].

α-Galactosylceramide (α-GalCer) is the prototypical NKT cell agonist [25]. It was discovered during a screen for anti-tumor agents derived from the marine sponge *Agelas mauritianus* [26], and is now widely used as a synthetic ligand because it activates both human and murine NKT cells. Following with the recognition of α-GalCer, NKT cells produce cytokines, undergo expansion, and subsequently activate NK cells, dendritic cells, B cells, and T cells [27–30]. Moreover, activated NKT cells induce cell death in tumor cells, like other cytotoxic cells, such as NK cells and cytotoxic T lymphocytes (CTL).

Several studies have sought to ascertain the role of NKT cells in modulating anti-tumor immune responses to B cell lymphomas [24,31–36]. While many of these studies have utilized established tumor models to examine the efficacy of autologous B cell lymphoma vaccines in combination with α-GalCer, the goal of this study was to evaluate NKT cell responses to B cell lymphomas, assess NKT cell function during lymphomagenesis, and determine the efficacy of α-GalCer in a spontaneous mouse model of B cell lymphoma in immunocompetent mice. We found that in the presence of an NKT cell agonist, both mouse and human NKT cells produce high levels of IFN-γ following recognition of malignant B cells; however, autologous NKT cell function diminishes during lymphomagenesis. Importantly, we found that treatment with a single dose of α-GalCer elicited effective anti-tumor immunity in a spontaneous mouse model of blastoid variant MCL.

## 2. Experimental Section

### 2.1. Peripheral Blood Mononuclear Cells (PBMC)

All donors gave written informed consent before enrolling in the study. The Institutional Review Board at the University of Maryland School of Medicine (UMSOM) approved this investigation. Peripheral blood was collected from patients undergoing treatment at the Marlene and Stewart Greenebaum Cancer Center at the UMSOM. The clinical diagnosis was confirmed in our patient population using cytogenetics. Data shown are from newly diagnosed patients prior to treatment. Peripheral blood mononuclear cells (PMBC) were also obtained from commercial vendors. Specifically, buffy coats were purchased from Biological Specialty Corporation and peripheral blood from two different, newly diagnosed MCL patients was purchased from AllCells, LLC (Alameda, CA, USA). PBMCs were isolated by Ficoll-Hypaque (Amersham Pharmacia Biotek, Uppsala, Sweden) density gradient centrifugation. Human primary B cells were isolated using the Pan B cell isolation kit from StemCell Technologies (Vancouver, BC, Canada) according to the manufacturer’s instructions. NKT cells were isolated and expanded as previously reported [37].

### 2.2. Mice

Wild-type C57BL/6 mice were purchased from The Jackson Laboratory (Bar Harbor, ME, USA). IL-14α transgenic mice and c-myc transgenic mice were generously provided by Dr. Julian L. Ambrus Jr. (State University of New York (SUNY) at Buffalo School of Medicine and Biomedical Sciences), and bred in specific pathogen-free facilities at the University of Maryland School of Medicine. All experiments were performed in accordance with procedures approved by the University of Maryland School of Medicine animal use and care committee. In order to generate the BV-MCL mouse model, we crossed c-myc transgenic (TG) mice with IL-14α TG mice to obtain double transgenic mice (DTG), as previously described [38]. Every DTG mouse is characterized by an initial leukemic phase and develops widespread lymphadenopathy and splenomegaly within three to four months of age. Isolation of liver MNC was performed as described previously [39]. Spleens and lymph nodes were harvested from tumor free and tumor-bearing mice, and processed into single-cell suspensions. Erythrocytes were lysed by hypotonic shock using ACK cell lysing buffer (Quality Biological, Inc., Gaithersburg, MD, USA). The remaining cells were washed twice with IMDM supplemented with 5% FBS (complete medium), then resuspended in the same medium.

### 2.3. Cell Lines

The Vα14^+^ NKT cell hybridoma cell lines DN32.D3 and N38-3C3 have been described [40–42] and were cultured in IMDM medium supplemented with 5% FBS, Pen/Strep and 2 mM L-glutamine. L-CD1dwt cells are CD1d1-transfected L cells, kindly provided by Dr. Randy Brutkiewicz (Indiana University School of Medicine, Indianapolis, IN, USA) and were cultured in DMEM supplemented with 10% FBS, 2 mM L-glutamine, Pen/Strep, and 500 μg/mL G418. Farage, a human B cell lymphoma line, was generously provided by Dr. Ronald Gartenhaus (University of Maryland School of Medicine, Baltimore, MD, USA); Mantle cell lymphoma lines, SP53 and JeKo-1 were kindly provided by Dr. Raymond Lai (University of Alberta, Edmonton, AB, Canada). Murine B cell lymphoma cell lines, WEHI-231, CH31, and CH33 were graciously provided by Dr. Gregory Carey (University of Maryland School of Medicine, Baltimore, MD, USA). The human B lymphoblastoid cell line transfected with human CD1d, C1R-CD1d, kindly provided by Dr. Mark Exley (Harvard Medical School, Boston, MA, USA), was used as a control. All B cell lymphoma lines were cultured in RPMI 1640 medium supplemented with non-essential amino acids (Sigma-Aldrich, St. Louis, MO, USA), sodium pyruvate (Gibco, Life Technologies, Carlsbad, CA, USA), vitamin solution (Gibco), 2-mercaptoethanol (Gibco), 10% fetal bovine serum (Gibco), and Pen/Strep (Gibco).

### 2.4. NKT Cell Assays

To measure NKT cell responses to B cell lymphomas, murine B cell lymphoma lines were incubated in the presence or absence of exogenous antigen (200 ng/mL), washed and cocultured (5 × 10^5^ cells/well) with the NKT cell hybridomas (5 × 10^4^ cells/well) in triplicate wells in 96-well microtiter plates. After a 20- to 24 h coculture, supernatants were harvested, and IL-2 was measured by ELISA, as it is the prototypical cytokine used to assess their activation [40,41]. To assess primary human NKT cell responses to B cell lymphomas, human B cell lines or primary B cells were incubated in the presence or absence of α-GalCer (100 ng/mL) and cocultured (10^5^ cells/well) with primary human NKT cells (2 × 10^4^ cells/well) in triplicate wells in 96-well microtiter plates. To assess endogenous primary murine NKT cell responses to B cell lymphomas in DTG mice following treatment with α-GalCer, splenocytes (5 × 10^5^ cells/well) were incubated in the presence or absence of α-GalCer (100 ng/mL) in triplicate wells in 96-well microtiter plates. After a 48 h coculture, supernatants were harvested, and IFN-γ and IL-4 were measured by ELISA. The limit of detection for each ELISA was <4 pg/mL.

### 2.5. FACS Analysis

Mouse B cell lymphoma lines were stained with PE-labeled mouse CD1d-specific antibody (clone 1B1, Biolegend, San Diego, CA, USA). Human B cell lines were analyzed for surface expression of CD1d by flow cytometry. Cells were stained with PE-labeled human CD1d specific clone CD45.2 (BD Biosciences, San Jose, CA, USA) or 51.1 (Biolegend, San Diego, CA, USA). Isotype control staining was performed to demonstrate specificity. Liver mononuclear cells were stained with anti-mouse TCRβ mAb (Biolegend, San Diego, CA, USA) and PBS57 loaded- (α-GalCer) CD1d tetramer (generously provided by the NIH tetramer facility, Emory University, Atlanta, GA, USA) to determine the percentage of NKT cells. Data were acquired using an LSR II (BD Biosciences, San Jose, CA, USA) and analyzed with FCS Express V3 (De Novo Software, Los Angeles, CA, USA).

### 2.6. Histological Analysis

Spleen sections were fixed in 4% paraformaldehyde (PFA) in phosphate-buffered saline (PBS) and sent to the Histology Core at the University of Maryland, Baltimore, for paraffin embedding and sectioning. Five-micrometer sections were prepared and used for hematoxylin and eosin (H&E) staining by the Histology Core Services (University of Maryland).

## 3. Results and Discussion

### 3.1. B Cell Lymphoma Lines Do Not Present an Endogenous Activating Antigen to NKT Cells

**Figure 1:**
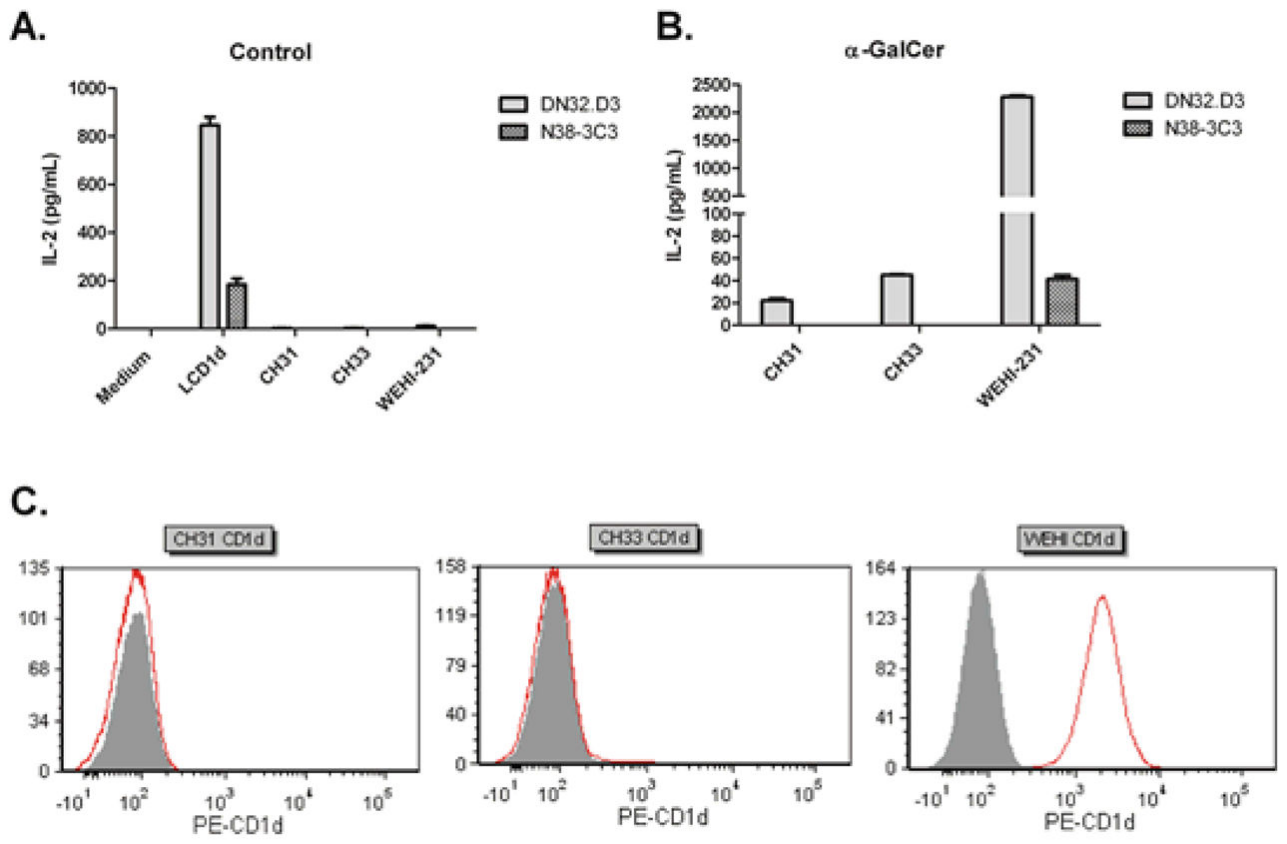
B cell lymphoma mediated activation of mouse NKT cell hybridomas. (**A**) Mouse B cell lymphoma lines (WEHI-231, CH31, and CH33) were cocultured with NKT cell hybridomas, DN32. D3 and N38-3C3. Recognition of CD1d was assessed by measuring IL-2 production in the supernatants by ELISA. L cells stably transfected with *mCD1d1* (LCD1d) served as a positive control; (**B**) B cell lines were pulsed with the potent NKT cell agonist, α-GalCer (200 ng/mL for 2 h), washed extensively with PBS and co-cultured with NKT cell hybridomas; (**C**) Cell surface CD1d expression on mouse B cell lymphoma lines. Murine B cell lymphoma lines were analyzed for surface expression of CD1d by flow cytometry. Cells were stained with PE-labeled mouse CD1d specific clone 1B1 (red histograms), the gray filled histograms indicate the isotype control.

It has been previously reported that murine splenocytes do not present an activating endogenous antigen to NKT cells [42]. However, it was unclear whether cellular alterations during malignant transformation could alter the lipid repertoire such that NKT cells would recognize B cell lymphomas. To determine if B cell lymphoma cells could directly induce NKT cell activation, we utilized a panel of murine B lymphoma cell lines. Mouse fibroblast cells stably transfected with *CD1d1*, LCD1d, were used as a positive control because these cells present an endogenous, activating antigen [41,43]. As shown in Figure 1A, it was found that, in the absence of exogenous antigen, there was minimal NKT cell activation by B cell lymphoma lines. However, the addition of the potent NKT cell agonist, α-Galactosylceramide (α-GalCer) resulted in differential activation of NKT cell hybridomas (Figure 1B). Interestingly, we found that culture with B cell lymphomas resulted in higher levels of activation by DN32.D3, compared to N38-3C3, suggesting some degree of variability within the NKT cell hybridomas. In addition, CD1d cell surface expression was highly variable in B cell lymphoma lines (Figure 1C). In CH31 and CH33 B cell lymphoma lines, CD1d expression was undetectable by flow cytometry, however, WEHI-231 cells expressed high levels of CD1d. Antigen-pulsed mouse B lymphoma cells stimulated cytokine production by NKT cell hybridomas. Taken together, these data demonstrate that the CD1d molecules expressed on the cell surface of the murine B cell lymphomas cells are indeed functional, however, B cell lymphomas do not present an endogenous, activating ligand.

### 3.2. Human NKT Cells Respond to B Cell Lymphoma in the Presence of α-GalCer

**Figure 2:**
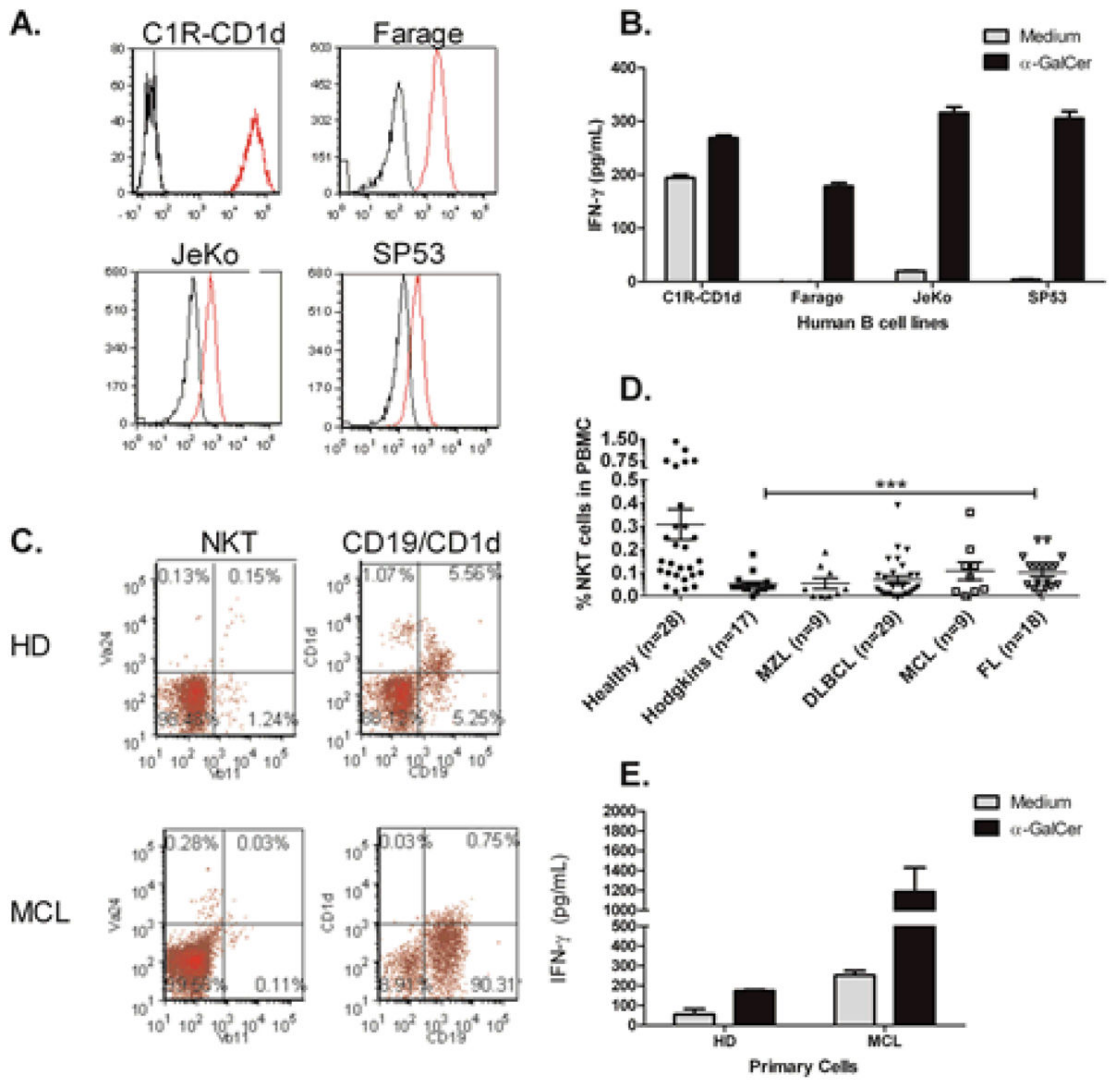
Healthy human NKT cells are stimulated by human B cell lymphomas, but circulating NKT cells are reduced in lymphoma patients. (**A**) Differential CD1d expression in human B cell lymphoma cell lines. Human B cell lymphoma cell lines were analyzed for surface expression of CD1d by flow cytometry. Cells were stained with PE-labeled human CD1d specific clone CD51.1 (red histograms). Isotype staining was performed to demonstrate specificity (black histograms). C1R-CD1d served as the positive control; (**B**) Primary human NKT cells were cocultured with human B cell lymphomas in the absence or presence of antigen- α-GalCer. Cytokine production following stimulation with C1R-CD1d served as the positive control; (**C**) Circulating NKT cells are reduced in MCL patients. PBMC were isolated from healthy donors (HD) and mantle cell lymphoma (MCL) patients and stained for flow cytometry; (**D**) Peripheral blood mononuclear cells (PBMC) were isolated from healthy donors and cancer patients. Cells were stained for Vα24^+^Vβ11^+^ TCR and analyzed by FACS. Scatterplots demonstrate the variation in the percentages of NKT cells. MZL-marginal zone lymphoma; DLBCL-diffuse large B cell lymphoma; MCL- mantle cell lymphoma; FL-follicular lymphoma. % NKT cells of healthy donors *vs.* lymphoma patients; Statistical analysis was performed using one way ANOVA *** *p* < 0.001; (**E**) Primary NKT cells expanded from a healthy donor were co-cultured with B cells isolated from a healthy donor or a mantle cell lymphoma (MCL) patient in the presence or absence of α-GalCer. Culture supernatants were harvested and ELISA was used to measure IFN-γ production. Data are representative of three independent experiments.

The level of CD1d cell surface expression on tumors is thought be directly correlated with NKT cell mediated cytotoxicity. High levels of CD1d expression result in higher tumor cell death, whereas low levels of CD1d expression result in minimal tumor cell lysis [44,45]. In addition, specific subsets of human B cells have been reported to express CD1d [46–48]. Therefore, we next examined CD1d expression on human B cell lymphoma cell lines (Figure 2A). It was found that CD1d is differentially expressed in B cell lymphomas. Specifically, there was high expression CD1d on Farage and intermediate expression on JeKo-1 and SP53 cells. C1R-CD1d cells have been stably transfected with hCD1d and served as positive controls for flow cytometry and for NKT cell activation. We have found that primary NKT cells can be activated by C1R-CD1d in the absence of exogenous antigen.

We next assessed whether CD1d expression on the B cell lymphomas correlated with CD1d-induced cytokine production by NKT cells. We found that in the absence of exogenous antigen (α-GalCer), human B cell lymphomas did not activate NKT cells (Figure 2B). The addition of α-GalCer, resulted in similar levels of IFN-γ induction by all of the B cell lymphoma lines examined, which did not directly correlate with CD1d expression (Figure 2A). This may be due to the potent activity of α-GalCer. When we compared CD1d expression on primary B cells in healthy donors and lymphoma patients, CD1d expression was highly variable. Our data suggest that presentation of α-GalCer by low levels of CD1d is sufficient to induce cytokine production by NKT cells. Importantly, it was found that while NKT cells are reduced in MCL patients, malignant B cells from an MCL patient stimulated high levels of IFN-γ production when incubated with primary NKT cells expanded from a healthy donor (Figure 2C–E). These data demonstrate that healthy NKT cells respond to malignant B cells and further suggest that appropriate modulation of NKT cells may enhance anti-tumor responses.

### 3.3. NKT Cell Responses Are Reduced during Lymphomagenesis

Our *in vitro* studies show that human NKT cells recognize B cell lymphomas. These data strongly suggest a role for CD1d-restricted NKT cells in cancer immune surveillance. There is a critical need for valid animal models in order to understand the role of NKT cells in cancer immunotherapy and develop novel, effective therapeutics. Ford *et al.* developed a spontaneous MCL mouse model for the aggressive blastoid variant of this disease (MCL-BV). It is generated by crossing IL-14α transgenic mice with c-myc transgenic mice [38], referred to here as double transgenic (DTG). C-myc is increased in most cases of MCL and c-myc transgenic mice (129 background) crossed with C57BL/6 wildtype have been shown to develop NKT cell responsive B cell lymphoma. The type of B cell lymphoma has been shown to be time dependent and is characterized as follicular to DLBCL (reviewed in [49]). Thus, we sought to use these spontaneous models of lymphoma to examine *ex vivo* NKT cell responses during lymphomagenesis.

**Figure 3:**
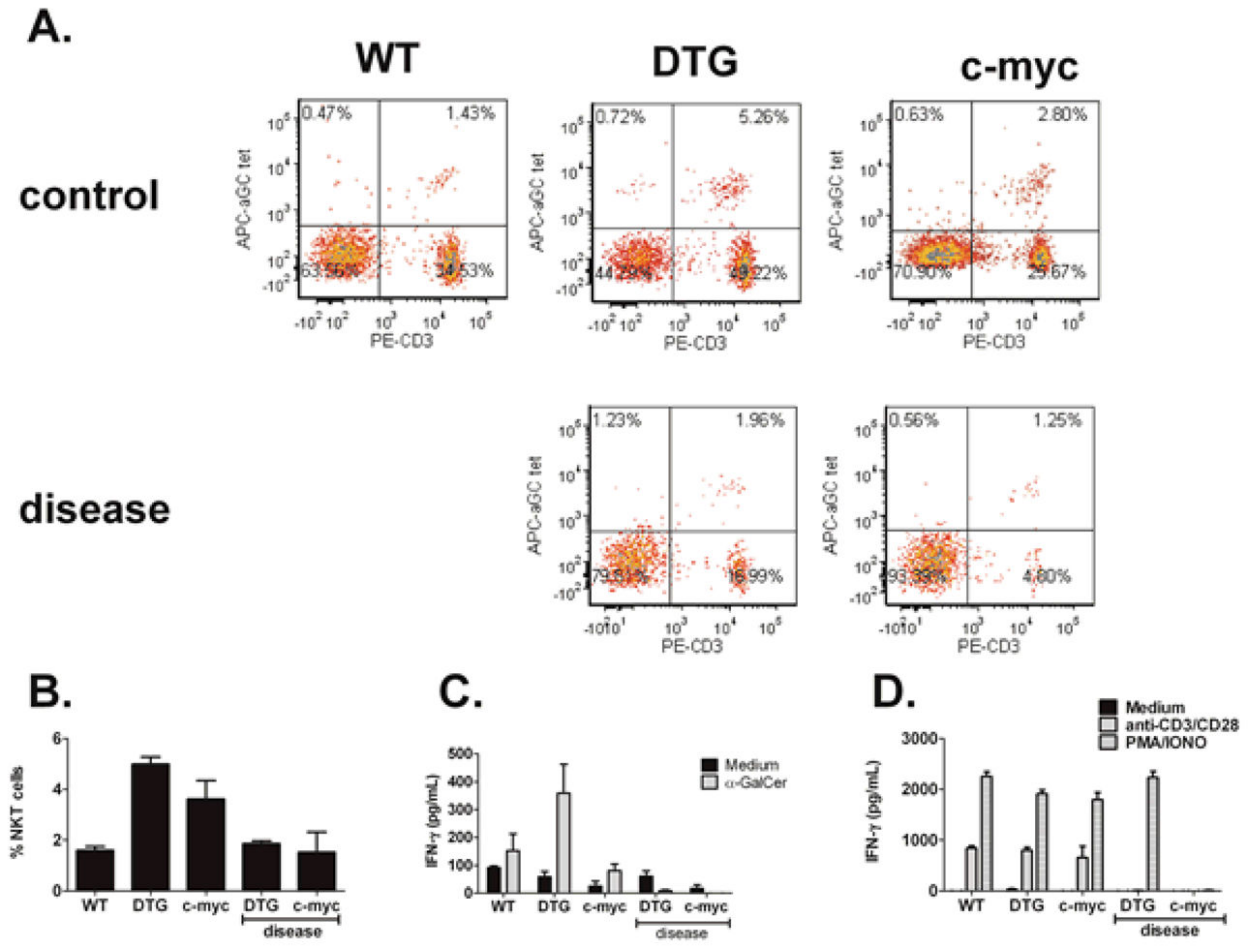
NKT cell responses are impaired during lymphoma progression. (**A**) Splenic NKT cell profiles in WT, DTG and c-myc Tg mice; (**B**) Splenic NKT cell % in WT, DTG, and c-myc Tg mice at six to seven weeks and 10–15 weeks of age. The term “disease” means that the mice were sick, as indicated by splenomegaly; (**C**) Primary mouse splenocytes cultured in medium alone or α-GalCer. After 48 h, IFN-γ levels were measured in the supernatant by ELISA; (**D**) To assess T cell function, splenocytes were cultured with anti- CD3/CD28 microbeads or PMA/ionomycin for 48 h. IFN-γ production in the culture supernatant was measured by ELISA. Data shown from one experiment and are representative of seven similar experiments.

To examine the role of NKT cells during lymphomagenesis, splenocytes were harvested from wildtype C57BL/6, tumor-free and tumor-bearing DTG and c-myc transgenic mice (Figure 3A–D). To assess the endogenous NKT response to the tumor, splenic leukocytes were cultured in medium in the presence or absence of α-GalCer. Supernatants were harvested 48 h later, and cytokine production was measured by ELISA. As shown in Figure 3C, mouse NKT cells respond to B cell lymphomas early during malignancy; however, following the establishment of large tumor masses as indicated by splenomegaly and lymphadenopathy, NKT cell responses are significantly reduced. In addition, we examined total T cell responses via stimulation of splenocytes with anti-CD3/CD28, and found that activation of conventional T cells was also lost during lymphomagenesis (Figure 3D). In these studies, PMA/ionomycin was used as a positive control.

### 3.4. Treatment of Tumor-Bearing Mice with α-GalCer Results in Reduced MCL Pathology

**Figure 4:**
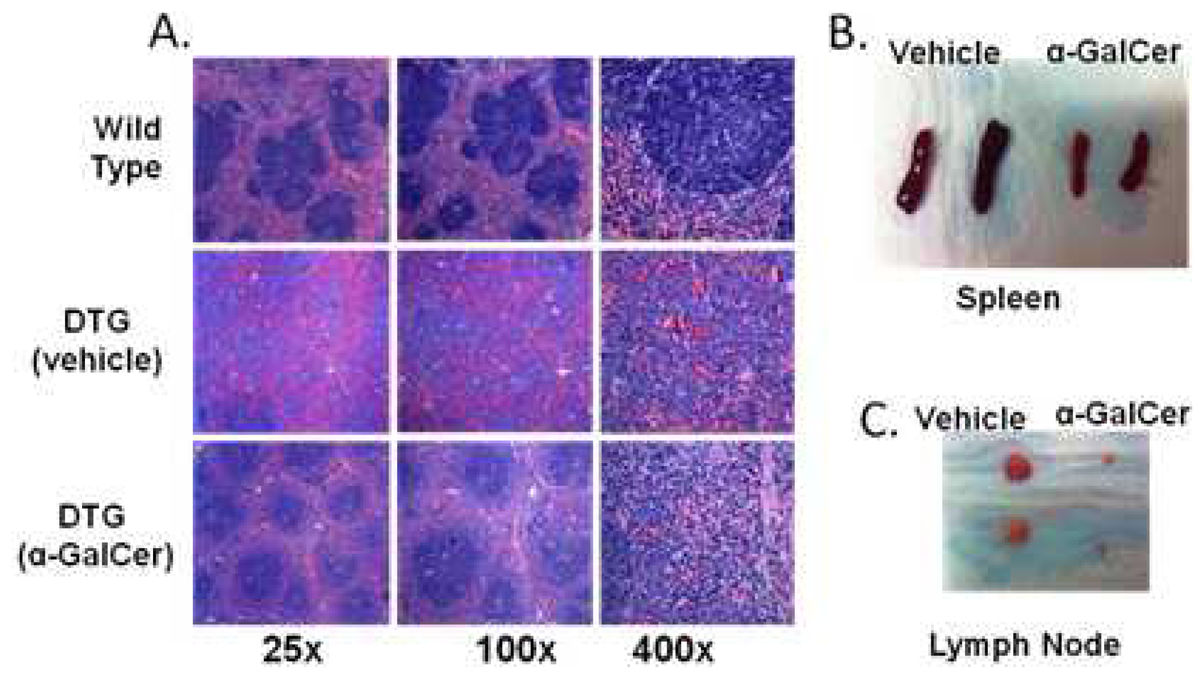
Activation of NKT cells reduces tumor burden *in vivo*. Eight-week old IL-14/c-myc double transgenic mice were treated with vehicle alone (DMSO) or α-GalCer (2 μg/mouse) in PBS i.v. and after six weeks, lymph nodes and spleens were harvested and examined for disease. (**A**) H&E staining of spleen sections shows improved splenic architecture and lower frequency of blastoid variant MCL cells as compared to vehicle treated controls; (**B**) spleens and (**C**) lymph nodes of α-GalCer treated mice show reduced splenomegaly and lymphadenopathy, respectively, as compared to vehicle-treated, littermate controls. These data are representative of three independent experiments of two to five mice per group; a total of 11 mice were treated with vehicle, and 10 were treated with α-GalCer.

The DTG mouse model is characterized by an initial leukemic phase and all of the mice have been reported to develop a B-cell malignancy within 4 months, which is preceded by high peripheral white blood cell counts that are 1.5 to 3 times higher than controls at two months [38]. Due to the high penetrance and high expression of Cyclin D1 (data not shown), which makes this model phenotypically similar to human MCL, we chose the DTG MCL-BV model to assess the therapeutic efficacy of NKT cell-based immunotherapy. In this study, eight-week-old DTG mice were injected with 2 μg α-GalCer (i.v.) or 0.02% DMSO in PBS (vehicle) and the mice were examined for disease six weeks post treatment. Littermates served as controls for each experiment. It was found that treatment with a single intravenous dose of α-GalCer was sufficient to delay disease progression *in vivo* (Figure 4A). Gross pathology clearly shows the effectiveness of activating NKT cells early during disease onset in order to delay tumor outgrowth (Figure 4B,C).

### 3.5. α-GalCer Stalls Progression of MCL-BV

**Figure 5:**
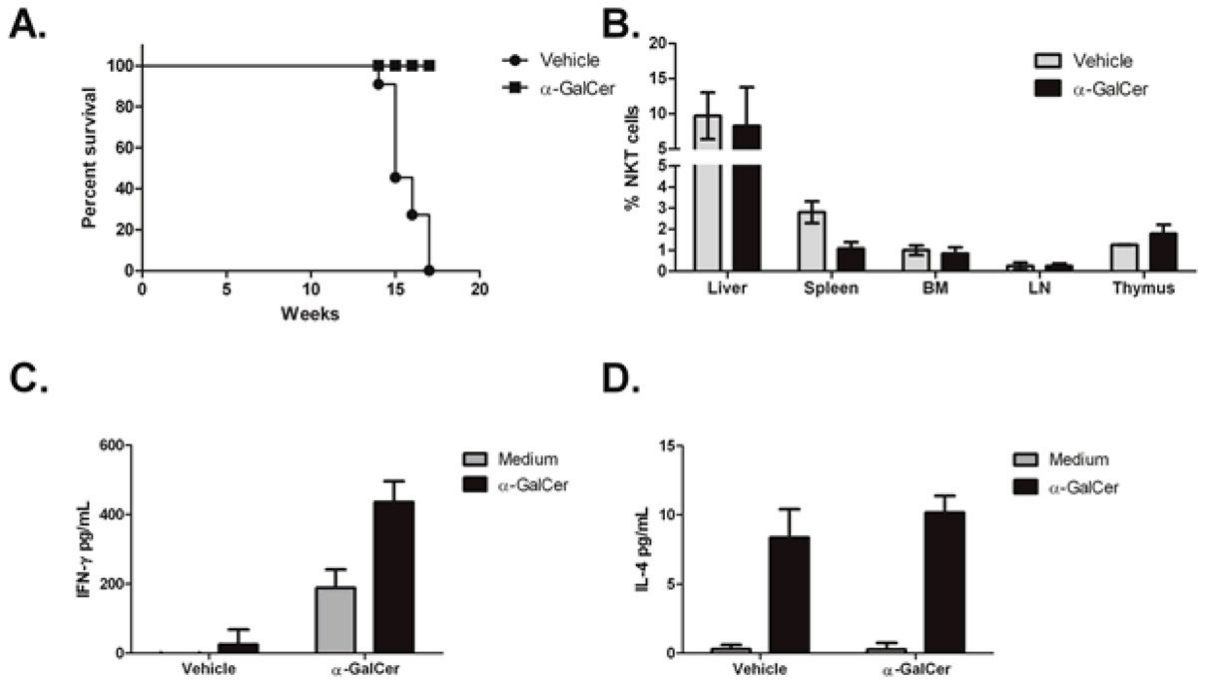
Activation of NKT cells with a single dose of α-GalCer (i.v.) increases survival in a mouse model of MCL-BV. (**A**) DTG mice were treated with α-GalCer or vehicle alone as described above. Survival curves showing mice treated with vehicle alone (*n* = 11) or with α-GalCer (*n* = 10). Mice were euthanized upon detection of severe lymphadenopathy along with their non-terminal counterparts; (**B**) Percentage of TCRβ^+^, PBS57 loaded (α-GalCer)-CD1d tetramer^+^ NKT cells in vehicle treated or α-GalCer treated mice; (**C**,**D**) Splenocytes were cultured for 48 h *ex vivo* in medium alone, or with α-GalCer for restimulation. Both baseline and restimulated levels were higher for (**C**) IFN-γ but not (**D**) IL-4 in mice treated with α-GalCer.

Several studies have shown that the use of immunomodulators is effective in controlling hematological malignancies by initiating or restoring the host anti-tumor immune responses to poorly immunogenic or immunosuppressive tumors. In a previous study, it was reported that a single therapeutic vaccination of irradiated, α-GalCer loaded autologous tumor cells was sufficient to inhibit the growth of established tumors [31]. In that study, the authors used an Eμ-myc transgenic tumor transplant model and demonstrated that anti-lymphoma immunity required NKT cells, NK cells, and CD8^+^ T cells. In the current study, we observed inhibition of disease development following a single injection of α-GalCer alone in a spontaneous mouse model of lymphoma. Survival curves were plotted based on severe disease, characterized by overtly visible lymphadenopathy, difficulty breathing, and lack of normal movement, indicating that the mice were terminal and had to be euthanized. Mice that did not show detectable lymphadenopathy upon outward examination were classified as non-terminal. We found that, at the indicated timepoints, vehicle treated mice showed severe terminal disease while their α-GalCer treated counterparts did not show any outward pathological manifestation of the disease (Figure 5A). These observations prompted us to examine the lymphocyte populations *ex vivo* to assess whether the treatment had an effect on the NKT cell population and to determine if the autologous NKT cells remained responsive to α-GalCer. Therefore, six weeks post-treatment, organs were harvested from 14-week-old DTG mice and NKT cell populations were assessed by flow cytometry and ELISA. No difference was observed in the percentage of NKT cells in the α-GalCer treated mice, compared to the controls (Figure 5B). To assess the immune response to NKT cell modulation, splenic leukocytes were cultured in medium in the presence or absence of α-GalCer. Supernatants were harvested 48 hours later, and cytokine production was measured by ELISA. We found that production of IFN-γ and IL-4 could be detected in splenocyte cultures following α-GalCer restimulation (Figure 5C,D). Higher levels of IFN-γ were produced by splenocytes from α-GalCer-treated mice, but there was no change in the production of IL-4. Overall, these data suggest that the activation of NKT cells can influence the host’s anti-tumor- response by producing and inducing other immune cells to produce high levels of IFN-γ.

## 4. Conclusions

Although MCL responds well to initial therapy, almost all patients relapse within 1–4 years even after intensive therapy. A more aggressive form, the blastoid variant presents at an earlier age and patients have a mean survival time of less than 20 months [2–4]. Therefore, the development of better treatment options is needed for this patient population. The goal of this study was to examine the efficacy of α-GalCer in inducing NKT cell responses to B cell lymphoma. We first performed *in vitro* studies to investigate NKT cell responses to both murine and human B cell lymphoma lines. It was found that the addition of α-GalCer to NKT cell/lymphoma co-cultures resulted in the induction of cytokine production by NKT cells, even when lymphoma CD1d expression was undetectable by flow cytometry. In primary cultures, human NKT cells produced higher levels of IFN-γ when cultured with MCL patient derived B cells, compared to B cells from a healthy donor indicating that there are mechanisms by which NKT cells recognize and respond to malignancy.

Given the anti-tumor role of NKT cells, we examined the percentage and *ex vivo* function of splenic NKT cells in two spontaneous, *myc* oncogene-driven mouse models of B cell lymphoma. It was found that prior to the development of splenomegaly, the percentage of splenic NKT cells was high and they were responsive to stimulation by α-GalCer. Treatment of DTG mice at early stages of disease with α-GalCer resulted in a significant reduction in disease pathology and the maintenance of IFN-γ producing NKT cells. Our data suggest that in treated mice, type I *i*NKT cells produce IFN-γ and can play an immunoregulatory role on the NK and CD8^+^ T cell populations by inducing cytokine production. Perhaps, in the absence of appropriate NKT cell activation; this regulation may not occur, resulting in lower IFN-γ production and poor anti-tumor immunity.

Similar to work by Mattarollo [31], in which they transplanted primary B-cell lymphomas derived from Eμ-myc transgenic mice into immunocompetent recipients and treated by vaccination with α-GalCer–loaded autologous tumor cells, we found that the activation of NKT cells resulted in significant inhibition of tumor growth and was associated with high levels of IFN-γ. In contrast to our study, this group did not see an effect when they treated with soluble antigen alone. This discrepancy may be due to the fact that in this model the tumors were injected, and control of this burden of lymphomas may require a more rapid induction of the immune response. In our spontaneous model tumors develop slowly over time, which may render them more sensitive to NKT cell responses.

Bjordahl *et al.* found that mice lacking *i*NKT cells were able to reject lymphoma [32], and proposed that this effect was due to tumor-specific CD8^+^ T cells being suppressed by *i*NKT cells. We believe that the role of iNKT cells is more nuanced. The tumor suppression in our mice may have arisen from the interplay between *i*NKT cells and CD8^+^ T cells, with the early activation of NKT cells facilitating the expansion of tumor specific CD8^+^ T cells; whereas, in their model, type I NKT cells may be playing an immunoregulatory role and that this model may induce a potent CD8^+^ T cell responses that *i*NKT cells are attempting to down regulate. In good agreement with our work, studies by Dong and colleagues showed that a single vaccination with α-GalCer loaded A20 lymphoma cells induced significant tumor regression in tumor bearing mice and elicited effective anti-tumor immunity against tumor challenge [33]. Their depletion and adoptive transfer studies implicated a role for CD4^+^ T cells in mediating anti-tumor immunity. It will be important in our future studies to clearly delineate the relative contributions of NK cells, NKT cells, CD4^+^, and CD8^+^ T cells. We are currently backcrossing each of the transgenic strains with CD1^−/−^ mice, so that we can further ascertain the role of NKT cells in MCL. Consistent with data reported by Renukaradhya *et al.* [34], we found that pro- and anti-inflammatory cytokines secreted by splenocytes from tumor-bearing mice correlated with tumor progression.

How do NKT cells regulate anti-tumor immunity and how does their activation result in a sustained increase in their number and function? Our data suggests that healthy primary human NKT cells specifically recognize and respond to B cell lymphomas, when appropriately stimulated. This results in higher levels of IFN-γ production, which is important for anti-tumor responses. α-GalCer-induced cytokine production, *ex vivo*, is also substantially greater in the transgenic mice early during malignancy, again demonstrating a level of control by NKT cells. We show that early NKT cell activation results in restored immune surveillance, and the host is able to delay tumor progression. Thus, in untreated mice, NKT cell function is impaired. Our work importantly demonstrates that during lymphomagenesis, NKT cells not only generate strong and effective responses, but also play a role in controlling the magnitude of the adaptive anti-tumor immune response and have a significant impact on disease outcome.
